# Portal biliopathy, magnetic resonance imaging and magnetic resonance cholangiopancreatography findings: a case series

**DOI:** 10.1093/gastro/gou062

**Published:** 2014-09-12

**Authors:** Ozdil Baskan, Cengiz Erol, Yusuf Sahingoz

**Affiliations:** ^1^Department of Radiology, School of Medicine, Istanbul Medipol University, Turkey and; ^2^Department of Radiology, Istanbul Medipol Mega University Hospital, Turkey

**Keywords:** biliary system, cavernous transformation, portal biliopathy, magnetic resonance imaging, magnetic resonance cholangiography

## Abstract

Portal biliopathy (PB) is a rare disorder, characterized by biliary ductal and gallbladder wall abnormalities seen in patients with portal hypertension. It most commonly occurs due to idiopathic extrahepatic portal vein obstruction (EHPVO). The abnormalities consist mainly of bile duct compression, stenoses, fibrotic strictures and dilation of both extrahepatic and intrahepatic bile ducts, as well as gallbladder varices. PB may mimic cholangiocarcinoma, sclerosing cholangitis, or choledocholithiasis. Misdiagnosis can be avoided using appropriate imaging modalities to prevent complications. We present the magnetic resonance imaging (MRI) and magnetic resonance cholangiography (MRCP) features of three patients with PB.

## INTRODUCTION

Portal biliopathy (PB) is defined as abnormalities of the extrahepatic and intrahepatic bile ducts, gallbladder and cystic duct, observed in patients with portal hypertension [1, 2]. Although jaundice and common bile duct (CBD) compression, associated with portal hypertension, was described by Fraser *et al.* [3] in 1944, the term ‘portal biliopathy’ was not used until the early 1990s [1].

PB is predominantly associated with extrahepatic portal venous obstruction (EHPVO). After a portal vein thrombosis, collaterals develop to bypass the obstruction, resulting in portal cavernoma. The peribiliary collateral vessels cause extrinsic compression, lead to reduction of the diameter of the intrahepatic and extrahepatic bile ducts. There are also underlying inflammatory changes, resulting in peribiliary fibrosis [4].

PB may mimic cholangiocarcinoma, sclerosing cholangitis, or choledocholithiasis [5, 6]. Misdiagnosis can be avoided by using appropriate imaging modalities to prevent complications. Magnetic resonance imaging (MRI) and magnetic resonance cholangiography (MRCP) can be used for diagnosis and differential diagnosis, and for determining the type of treatment required. We herein present the MRI and MRCP features of three patients with PB.

## CASE PRESENTATION

### Case 1

A 40-year-old man with a history of hyperhomocysteinemia, and who had portal vein thrombosis for 2 years, presented with abdominal pain, fever, nausea and vomiting for the previous 3–4 weeks. His physical examination was unremarkable. He had jaundice but signs of decompensated liver disease were absent. Viral profiles, including hepatitis B and C, were negative. The laboratory test results were total protein 6.6 g/dL, albumin 3.3 g/dL, total bilirubin 5.2 mg/dL, aspartate aminotransferase (AST) 46.5 U/L, alanine aminotransferase (ALT) 76.6 U/L and γ-glutamyl transferase (GGT) 135 U/L. Ultrasonography revealed dilation of the intrahepatic bile ducts; the portal vein could not be seen and was replaced by anechogenic tubular structures. Doppler ultrasound confirmed cavernomatous transformation of the portal vein (not shown).

**Figure 1:**
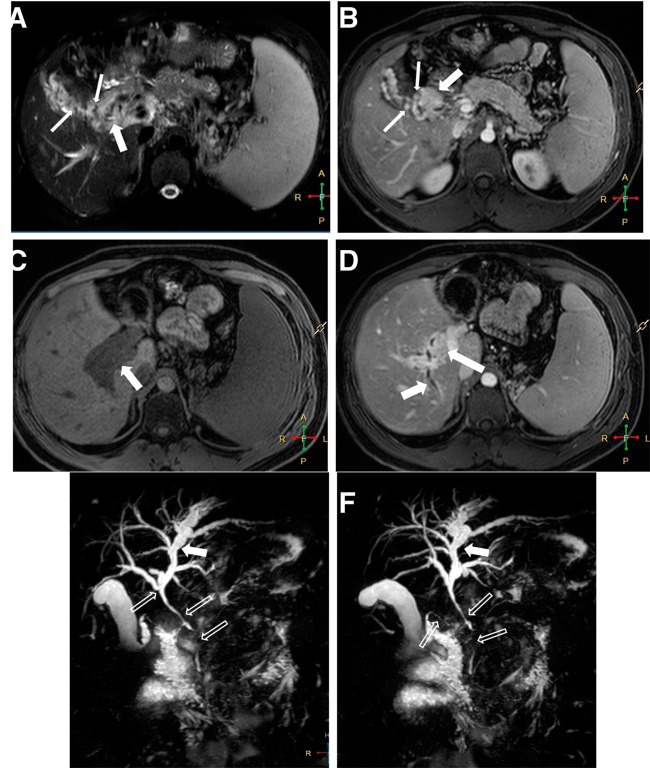
Focal and circumferential wall thickening with enhancing foci, suggesting epicholedochal veins and fibrosis (thick arrows), gallbladder varices (thin arrows) appear as low signal intensity on fat saturated T2-weighted imaging (A) and as enhancing on contrast-enhanced fat-saturated T1-weighted image (B). Biliary ductal wall thickening indicates hypointensity on fat-saturated T1-weighted imaging (C) and enhancement on fat-saturated imaging (B, D) (thick arrows). MRCP images (E, F) show multiple biliary stenoses involving the common bile duct (CBD) and proximal right hepatic duct are seen with proximal bile duct dilation (open arrows). Axial fat-saturated T2-weighted imaging (A), axial contrast-enhanced fat saturated T1-weighted imaging (B) at the same level with (A), axial fat-saturated T1-weighted (C), axial contrast-enhanced fat saturated T1-weighted imaging (D) at the same level with the (C), and MRCP images (E and F).

MRI demonstrated intrahepatic biliary dilation (Figure 1); the portal vein was not visualized separately. The portal vein showed cavernomatous transformation that encircled the CBD, proximal intrahepatic bile ducts and the gallbladder was characterized by focal and circumferential wall thickening, suggesting epicholedochal veins and fibrosis. Biliary ductal wall thickening indicated hypointense on fat-saturated T1-weighted imaging (Figure 1C) and enhancement on fat-saturated imaging (Figures 1B and 1D). MRCP images (Figures 1E and 1F) showed multiple biliary stenoses involving the CBD and proximal right hepatic duct, with proximal bile duct dilation. There were no focal lesions or changes suggestive of chronic liver disease. The patient was followed-up in outpatient clinics. The patient was re-admitted on several occasions because of recurrent cholangitis.

### Case 2

A 38-year-old man, with a one-year history of myeloproliferative disorder, presented to our hospital with abdominal pain. Physical examination was unremarkable. Laboratory tests showed mild liver abnormalities. The laboratory test results were total protein 6.2 g/dL, albumin 3.66 g/dL, total bilirubin 3.5 mg/dL, AST 52.9 U/L, ALT 92.6 U/L, lactate dehydrogenase (LDH) 260 U/L, GGT 119 U/L, C-reactive protein (CRP) 23.66 mg/dL, activeated partial thromboplastin time (APTT) 39.4 s, prothrombin time (PT) 17 s, PT-INR (international normalized ratio) 1.9 and haemoglobin 13.2 g/dL. Ultrasound subsequently revealed thrombosed portal and splenic veins, with multiple anechogenic tubular structures, at the *porta hepatis* and moderate intrahepatic biliary dilation (not shown).

**Figure 2:**
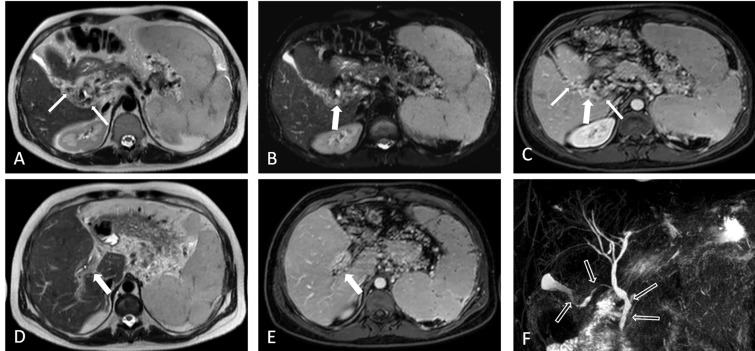
Focal and circumferential wall thickening with enhancing foci, suggesting epicholedochal veins and fibrosis (thick arrows), gallbladder varices (thin arrows) appear as low signal intensity on axial T2-weighted (A) and fat-saturated T2-weighted images (B), and as enhancing on contrast-enhanced fat-saturated T-1 weighted imaging (C). Biliary ductal wall thickening indicates hyperintensity on T2-weighted imaging (D) and enhancement on fat-saturated T1-weighted imaging (E) (thick arrows). MRCP imaging (F) shows multiple stenoses, and the gallbladder wall irregularities (open arrows). Axial T2-weighted imaging (A), axial fat-saturated T2-weighted imaging (B), axial contrast-enhanced fat saturated T1-weighted imaging (C) at the same level; axial T2-weighted (D), axial contrast-enhanced fat saturated T1-weighted imaging (E) at the same level; and MRCP imaging (F).

MRI demonstrated mild intrahepatic bile duct dilation (Figure 2). The splenic and portal veins were not visualized separately. Cavernomatous transformation was detected. Collateral vascularization encircled the CBD, common hepatic and cystic ducts and the gallbladder, characterized by enhancing focal and circumferential wall thickening (Figure 2C) suggesting epicholedochal veins and fibrosis. Biliary ductal wall thickening showed as enhancement on fat-saturated imaging (Figure 2E). MRCP images (Figure 2F) show multiple mild biliary stenoses involving the CBD and distal common hepatic duct. Cystic ductus and gallbladder contour irregularities were demonstrated. The patient was treated for his primary disease. He is currently asymptomatic.

### Case 3

A 13-year-old girl presented to our hospital, with haematemesis and melena. Physical examination and laboratory findings were unremarkable. She had developed melena when she was 3 years old. She had a history of umbilical venous catheterization in the neonatal period. Her developmental history was also reported to be normal until the age of seven, when she started to show growth retardation. There was no family history of such a problem. Laboratory findings were total protein 7.21 g/dL, albumin 4.16 g/dL, total bilirubin 0.4 mg/dL, AST 18.4 U/L, ALT 9.6 U/L, CRP 0.71 mg/dL, PT 15.1 s, PT-INR 1.27 and haemoglobin 9.2 g/dL. Ultrasound at that time showed features of chronic liver disease with cavernous transformation (not shown).

**Figure 3:**
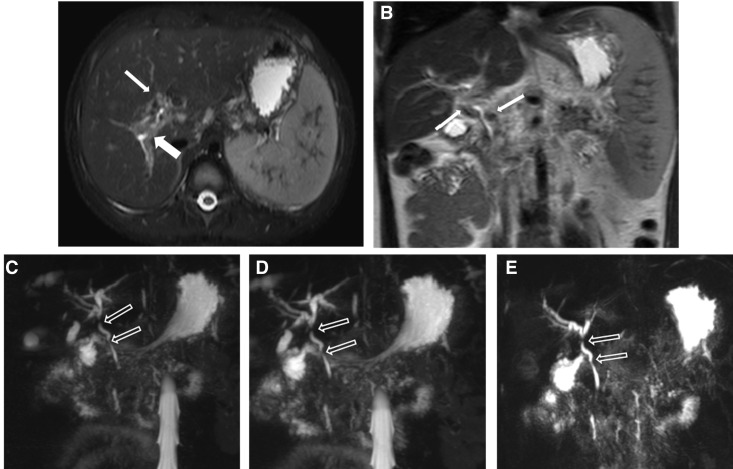
A concentric low–signal intensity area is seen surrounding the bile ducts, suggesting epicholedochal veins and fibrosis on axial fat-saturated T2-weighted imaging (A) (thick arrows), corresponding to signal-void collateral vessels (thin arrows) on axial fat-saturated T2-weighted (A) and coronal T2-weighted images (B). MRCP images (C–E) demonstrating narrowing of the common bile duct (CBD) and common hepatic duct (open arrows), with bile duct dilation. Axial fat-saturated T2-weighted imaging (A), coronal T2-weighted image (B), MRCP images (C, D, E).

MRI showed a concentric low-signal intensity area surrounding the bile ducts on axial fat-saturated T2-weighted imaging, suggesting epicholedochal veins and fibrosis, and signal-void collateral vessels on axial fat-saturated T2-weighted and coronal T2-weighted imaging (Figures 3A and 3B). MRCP images showed narrowing of the CBD and common hepatic duct, with bile duct dilation secondary to compression of the distal and proximal CBD and proximal common hepatic duct by these varices (Figures 3C, 3D and 3E). Features of chronic liver disease with splenomegaly were also noted. Follow-up of the patient was not available as the patient continued her theraphy in another hospital.

## DISCUSSION

PB is an uncommon manifestation, used to describe changes in the bile ducts due to cavernous transformation in patients with portal hypertension [2]. PB may lead to hepatic dysfunction if not treated [7]. This disorder is most commonly associated with EHPVO. Studies have shown that changes in the bile ducts occur in 81–100% patients, although only 5–30% have symptoms of biliary obstruction [4, 5, 8]. In a small proportion of patients, the condition has also been described in non-cirrhotic portal fibrosis and cirrhosis, [5].

Causes of EHPVO include clotting and myeloproliferative disorders, neonatal umbilical vein catheterization, abdominal post-operative complications, dehydration, intra-abdominal inflammatory diseases, and direct invasion or extrinsic compression by tumours. Our cases had known risk factors, as mentioned above. The drainage veins ascend along the course of the CBD and form epicholedochal and paracholedochal venous plexuses. The epicholedocal plexus lies on the surface of the bile duct and forms a fine reticular venous plexus not greater than 1 mm in diameter. The paracholedochal plexus runs parallel to the CBD. After portal vein thrombosis, new collaterals develop, resulting in portal cavernoma. The dilated collateral plexuses cause extrinsic compression—smooth indentations in the biliary ducts—which can progress to narrowing and stenosis. There are also inflammatory and ischaemic changes underlying the portal thrombosis, resulting in peribiliary fibrosis [1, 4]. Development of varices in the gallbladder wall as a collateral pathway via the cystic vein is also a characteristic feature of PB [2] (Figures 1 and 2).

PB has a slowly progressive character. However, patients with symptomatic PB are normally older than those presenting with EHPVO [4]. Symptoms may occur if high-grade obstruction of the bile ducts develops, and include right upper quadrant pain, jaundice, pruritus, cholestasis, and cholangitis. Patients with long-term obstruction or inadequate endoscopic or surgical management may develop secondary biliary cirrhosis (2–4% of cases) [2].

Recent advances in the spatial and temporal resolution of MRI permit excellent demonstration of biliary system anatomy. MRI has replaced endoscopic or direct cholangiography (DC) as the diagnostic procedure of choice for numerous conditions involving the biliary system, including PB. This has limited the role of direct or endoscopic cholangiography for therapeutic purposes [9]. MRCP identifies all the morphological changes of the biliary system, similar to the DC. Dynamic, contrast-enhanced MRI allows anatomical relation of biliary strictures to peribiliary enlarged vessels, which provides additional information beyond cholangiographic studies. MRI is also helpful in differential diagnosis, for example cholangiocarcinoma. The imaging features of PB on MRCP include biliary strictures, dilations, a wavy appearance of the bile ducts and varicose veins located at the ductal walls and gallbladder.

Shin *et al.* classified the MRI features of the PB patients into three types—as varicoid, fibrotic, or mixed—depending on the appearance of the bile duct at the point of obstruction [9]. The investigators in these studies proposed that varicoid PB is a biliary obstruction by large collateral (paracholedochal) veins that compress and distort the extrahepatic bile duct, while fibrotic PB results from smaller intramural (epicholedochal) collaterals visible as narrowed, thickened, and densely enhancing bile ducts. The importance of this distinction is that varicoid PB may be reversible with decompression of the collateral veins, while the fibrotic type is not [10]. In our case series, MRI and MRCP findings of the case 1 and 2 were consistent with fibrotic type (Figures 1 and 2) and the findings of the third case were consistent with the mixed type (Figure 3).

Cavernous transformation of the portal vein due to EHPVO is not infrequent, but biliary obstruction in association with this disorder is distinctly uncommon. Early diagnosis and treatment are very important for these patients, because a prolonged biliary duct obstruction can lead to the development of secondary biliary cirrhosis. MRI and MRCP can be used for diagnosis, differential diagnosis and follow-up.

**Conflict of interest:** none declared.
